# Integrating YOLOv8-based disaster detection with deep reinforcement learning for closed-loop UAV emergency response scheduling: A simulation study

**DOI:** 10.1371/journal.pone.0361374

**Published:** 2026-10-29

**Authors:** Chunsheng Zhang, Ruichun He

**Affiliations:** School of Traffic and Transportation, Lanzhou Jiaotong University, Lanzhou, China; Xidian University, CHINA

## Abstract

Aerial perception and dispatch scheduling in unmanned aerial vehicle emergency response are commonly arranged as a one-directional pipeline, and the contribution of closing the loop between them has not been measured under controlled conditions. This study evaluates a framework in which a YOLOv8 scene classifier gates task discovery, a Disaster Situation State Matrix aggregates spatially indexed detection outputs under exponential temporal decay, and a proximal policy optimization scheduler acts on that matrix. Evaluation used a 32 × 32 grid testbed with four vehicles, ten training seeds per configuration, and one hundred frozen evaluation scenarios per seed, with a complete two-by-two ablation separating policy access to the state matrix from the rate at which the matrix is refreshed. Runtime perception updating raised task completion from 0.4971 to 0.5859 relative to an initial-snapshot policy, a gain of 8.87 percentage points (95% CI 7.26 to 10.32, Holm-adjusted p = 0.0078). Policy access to the state matrix carried a main effect of 36.42 percentage points (95% CI 34.83 to 37.79) and refresh rate a main effect of 1.81 percentage points (95% CI 0.90 to 2.82), with no evidence of interaction. Under a shared six-action interface and identical perception constraints, the proposed scheduler exceeded a greedy heuristic by 3.44 percentage points (95% CI 2.03 to 4.91, Holm-adjusted p = 0.0078). Replacing confusion-matrix sampling with replayed YOLOv8 predictions on held-out images reduced completion by 1.28 percentage points, and temporally persistent detection errors extended the longest uninterrupted error run to seventy-one steps. Structural exposure of the state matrix to the scheduling policy, rather than refresh frequency alone, accounts for the observed performance of the framework.

## 1 Introduction

Rising occurrences of both natural and artificial disasters pose greater challenges to emergency services. Aerial platforms assess damaged infrastructure and build a picture of the affected environment. A central operational problem in this setting is scheduling multiple vehicles against disaster information whose value varies across locations and over time. Reinforcement-learning approaches outperform purely heuristic dispatch, yet few studies identify which design element produces the gain [1]. Recent surveys cover the operational challenges of unmanned aerial vehicles across payload, autonomy, regulation and emerging applications [2]. High-resolution imagery captured shortly after an event supports structural triage, demonstrating the practical value of rapid aerial assessment [3].

Recent contributions approach the problem from several directions. Joint optimization of flight, task offloading and resource allocation reconciles energy against quality-of-service constraints in emergency response [4,5], while learning-based trajectory design addresses partial observability in disaster-area communications through multi-agent policy optimization, imitation from expert demonstrations, and federated coordination across heterogeneous devices [6–8]. Latency-aware scheduling and energy-constrained variants extend the same formulation to settings where computation migrates to the network edge or where user-equipment power is scarce [9,10]. Planning research has established that coverage, obstacle avoidance and battery limits can be handled within a single sequential decision framework, whether as constrained tour optimization, goal-conditioned decomposition, hybrid metaheuristics, or scalable value-based control [11–16]. Aerial perception has advanced along a parallel axis, improving recognition of small or visually degraded targets through transformer-enhanced backbones, lightweight architectures, multi-scale fusion and complementary sensing [17–21]. Multi-agent scheduling has concentrated on partial observability arising from communication and coordination, supplying the environment state directly to the policy [22–28]. These contributions share one architectural feature: the layer that infers disaster state from imagery is separated from the layer that selects dispatch actions. Planning proceeds on a map supplied in advance, detection is optimized without reference to the decision that consumes it, and the perceptual state evolving as the platform traverses the scene is treated as an environmental nuisance rather than as a feedback channel the policy could exploit.

Integration of the two layers remains less developed than either layer alone. Target assignment has been combined with dynamic obstacle perception in multi-UAV planning, maintenance policies have been learned over partially observable states that are difficult to estimate, aerial and ground decision processes have been coupled in joint courier scheduling, and velocity-obstacle formulations have achieved perception-coupled reactive control for surface and micro aerial vehicles [29–33]. A separate line of work has long held that sensing is itself an action to be planned, not a passive input, developing this position from early formulations of active perception through its contemporary restatement [34,35], through view planning that selects where to look next [36], and through active mapping in which the trajectory is chosen to reduce uncertainty about the environment [37]. Partially observable formulations provide the corresponding formal account, in which an action alters not only the physical state but also the information available at subsequent decisions [38]. What that literature has established for localization, mapping and object recognition has not been carried over to dispatch scheduling, where the quantity acted upon is not the accuracy of a reconstruction but the set of tasks the scheduler is able to reach. These systems merge perception and decision at the level of outputs, where a detection result triggers or modifies an action, rather than at the level of representation, where an aggregated perceptual state persists across time and conditions the policy. The distinction matters because only the latter arrangement makes the informational consequence of a trajectory available to the decision that produced it, and its contribution has not been isolated under controlled conditions.

This study treats perception and action as components of a single closed loop, in which detection outputs continuously update a spatially indexed representation of disaster state, that representation enters the scheduling policy observation at every decision, and the resulting trajectories determine which cells receive subsequent visual coverage. Coupling is the experimental variable, so that the contribution of feedback between perception and action can be separated from those of detection quality, policy design and reward shaping. All configurations share one action interface, one perception constraint and one training budget, so that the estimated quantities are the separate contributions of policy access and refresh rate, a decomposition that prior perception-action architectures have not isolated under matched conditions.

The framework combines a YOLOv8 scene classifier that gates task discovery, a Disaster Situation State Matrix that couples exponential temporal decay with assimilation of detection outputs at observed cells, and a proximal policy optimization scheduler conditioned on that matrix. Evaluation proceeds through a complete factorial ablation separating policy access to the matrix from its refresh rate, comparison against matched algorithmic baselines under a shared action interface and perception constraint, characterization of robustness across three structurally distinct error channels together with replayed per-image predictions, and component-level timing of the perception and scheduling paths.

## 2 Methods

### 2.1 Closed-loop system architecture

This study formulates UAV emergency response as a partially observable sequential decision problem in which aerial perception and dispatch scheduling form a coupled closed loop. Three modules constitute the architecture: a YOLOv8-based aerial classifier that infers disaster type from UAV camera observations, a Disaster Situation State Matrix (DSSM) that aggregates spatially indexed detection outputs across time, and a Proximal Policy Optimization (PPO) scheduling policy that issues dispatch actions conditioned on the DSSM. Information flows in both directions: detection outputs refresh the DSSM, the scheduling policy reads the DSSM as part of its observation, and the resulting trajectories determine which cells receive subsequent coverage and hence which detections follow.

Operation proceeds in discrete time. At each step, the UAV observes the cell at its current position, the detection module produces a classification output, this output triggers a DSSM update, the scheduling policy selects an action based on the augmented state that includes the DSSM, and the environment transitions to the next configuration determined by both UAV motion and the underlying disaster evolution. This formulation captures the essential property that perception and action are temporally interleaved within a single closed loop, in contrast to open-loop variants where detection occurs only at the initial time step or where detection outputs do not enter the scheduling policy state. Fig 1 presents the architectural decomposition.

**Fig 1:**
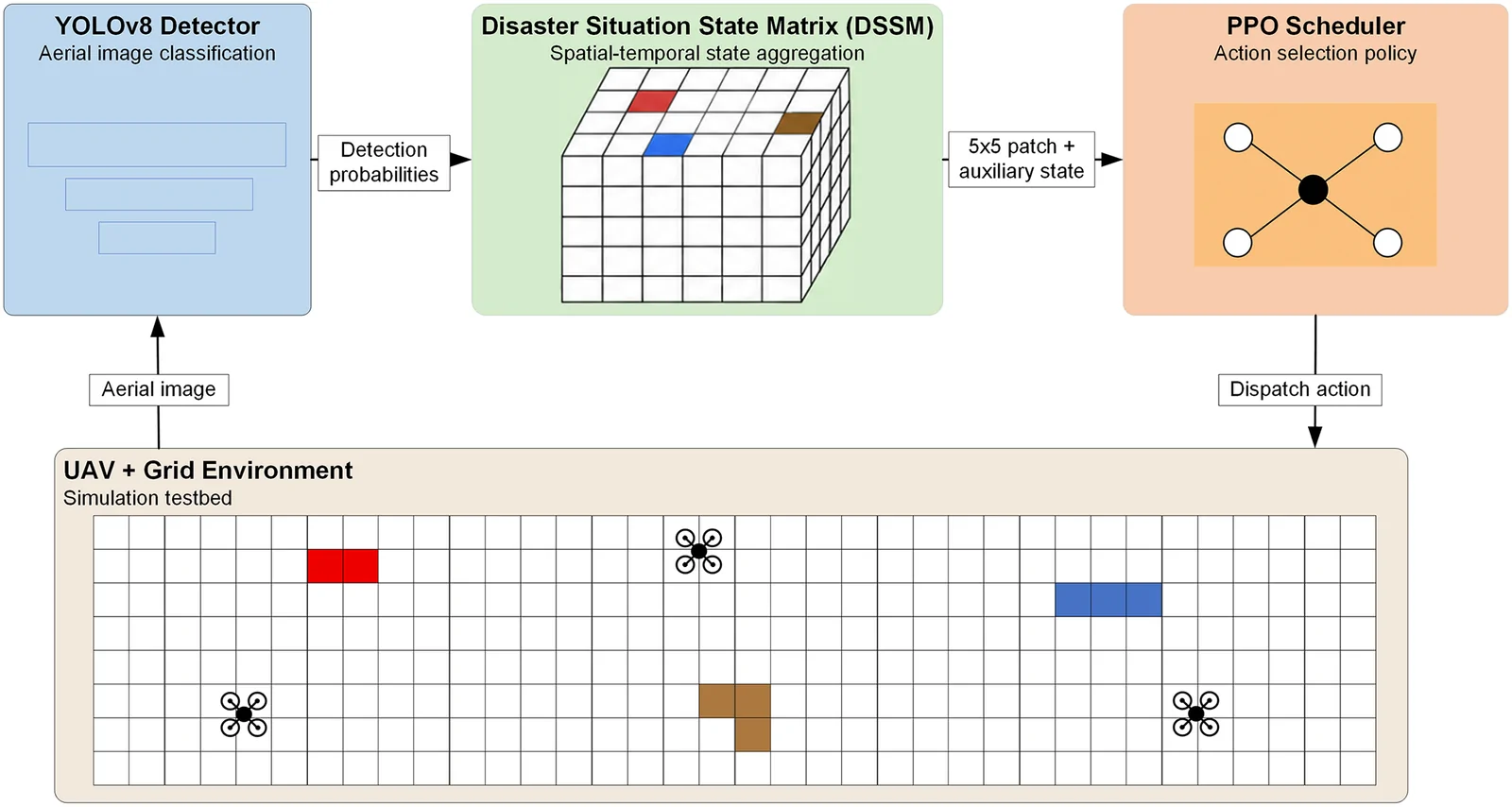
Closed-loop architecture integrating YOLOv8-based disaster detection, the Disaster Situation State Matrix, and PPO-based scheduling.

Fig 1 shows the feedback path along which detection outputs refresh the state matrix, the scheduling policy reads it, and the resulting trajectories determine which cells receive subsequent coverage.

### 2.2 Simulation environment design

The simulation environment is a two-dimensional grid on which vehicle motion, disaster evolution and demand-point service proceed as discrete state transitions. Each cell encodes its ground-truth disaster class, current severity level, and service status, while a road-network adjacency structure constrains UAV transitions to topologically valid moves between neighboring cells. The environment abstracts flight dynamics and represents motion as discrete transitions on a lattice, so that vehicle-level control and sensor noise do not enter the comparisons reported here.

UAV state at each step comprises position, remaining battery, and an internal target indicator, while the discrete action set comprises six options corresponding to motion in the four cardinal directions, a stationary hover, and a return-to-base command. Each cell traversal consumes a unit of battery, with the per-episode budget calibrated such that the optimal-policy energy expenditure occupies the upper half of the available budget, so that energy binds throughout the episode and never becomes a slack variable for the scheduling policy. Disaster evolution follows a deterministic spatial diffusion script that introduces new disaster zones at seeded random times and increases severity in unmitigated zones following a logistic growth profile, ensuring temporal non-stationarity that necessitates ongoing detection.

Task discovery is gated by perception throughout. A demand point becomes eligible for service only after the corresponding cell has been observed and assigned a disaster class by the detection module, so that ground-truth demand locations and release times are never supplied to the scheduler. This constraint applies identically to every configuration and to every algorithmic baseline, and it makes detection a precondition for service instead of an auxiliary signal.

The main experimental evaluation uses a 32 × 32 grid with thirty-five demand points and three base stations, illustrated in Fig 2.

**Fig 2:**
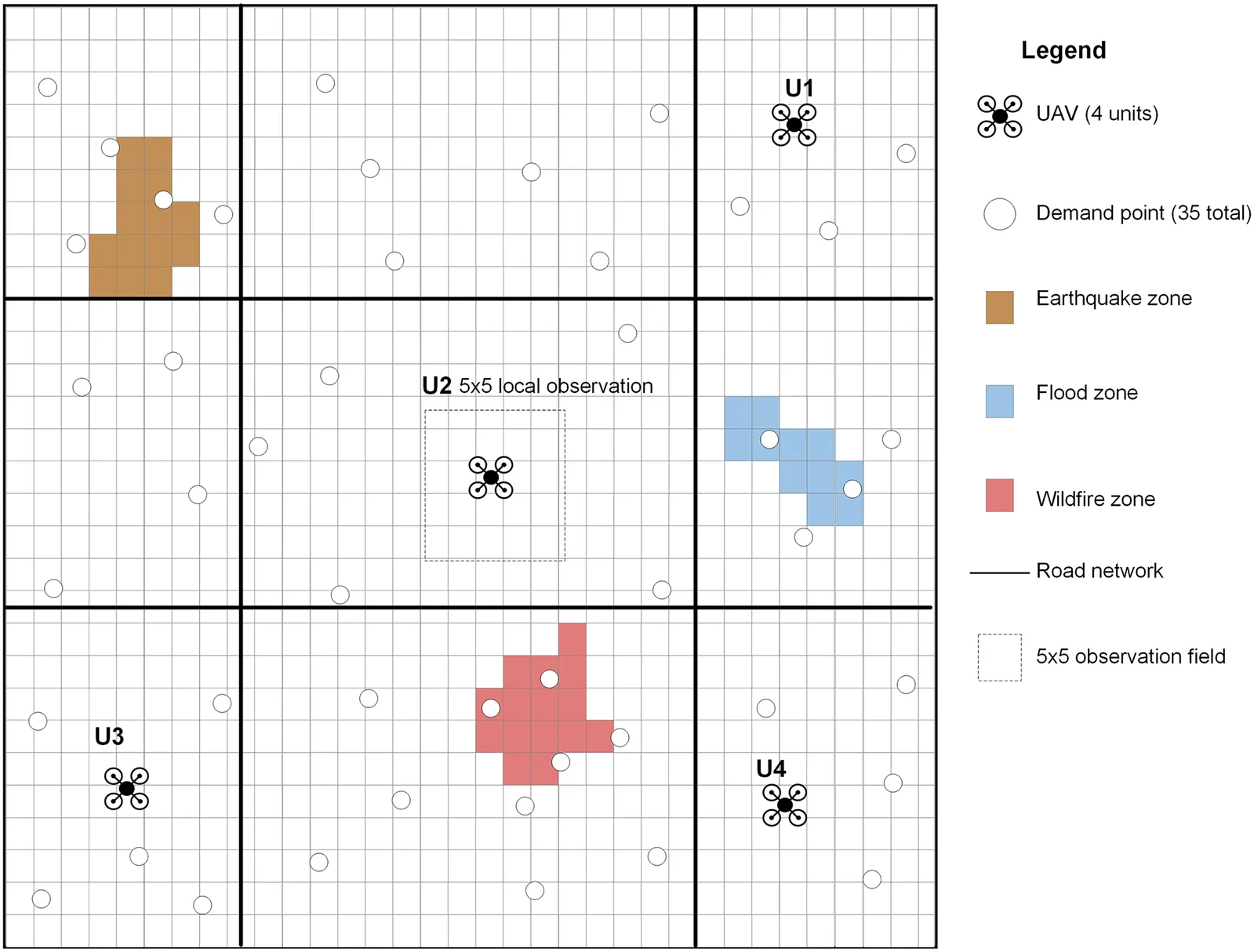
Two-dimensional grid-based simulation environment.

Fig 2 renders road-network connectivity as edges and demand points as marked locations, with color intensity in disaster zones encoding severity level. Dispersion of demand points relative to base stations illustrates the geometric difficulty that the scheduling policy must navigate. Complete numerical specifications governing the environment, detection module, and learning algorithm are consolidated in Table 1.

**Table 1:** Specification of simulation, detection, and learning components.

Component	Parameter	Value
Environment	Grid size	32 × 32
Environment	Demand points	35
Environment	Number of UAVs/bases	4/3
Environment	Battery capacity	200 per UAV
Environment	Disaster classes	4 (earthquake, flood, wildfire, normal)
Environment	Episode horizon	500 steps
Environment	DSSM observation field	5 × 5 cells centered on UAV
Environment	Action space	N, S, W, E, hover, return-to-base
YOLOv8	Primary backbone	yolov8s-cls
YOLOv8	Additional backbones	yolov8n-cls, yolov8m-cls
YOLOv8	Input resolution/epochs	96 × 96/50
YOLOv8	Optimizer/learning rate	AdamW/0.001
PPO	Implementation	Stable-Baselines3 2.8.0 + sb3-contrib
PPO	Learning rate/clip ratio	$3\times 10^{-4}$ /0.20
PPO	GAE/discount factor	0.95/0.99
PPO	Batch/rollout/epochs	256/512/5
PPO	Training steps/parallel environments	140,000/4
PPO	Normalization	Frozen per-model VecNormalize
Reward	Default weights	(1.0, 1.0, 1.0)
DSSM	Assimilation weight/decay rate	0.70/λ=0.00505 (per-step persistence 0.994962)
Random seeds	Training	31–40
Random seeds	Evaluation	2001–2100
Statistics	Independent unit	Training seed
Statistics	Inference	Paired bootstrap CI, exact Wilcoxon, Holm

Parameter values in Table 1 combine Stable-Baselines3 PPO defaults with task-specific calibrations established during pilot runs.

### 2.3 Disaster detection module

Detection is handled by yolov8s-cls, a YOLOv8 variant configured for whole-image scene classification instead of bounding-box localization [39]. This choice aligns with the cell-level granularity of the DSSM: each UAV observation corresponds to the aerial view of one grid cell, and the classifier assigns one of four labels (earthquake, flood, wildfire, or normal). Training uses the publicly available AIDERv2 dataset [40], partitioned at a 80.1/10.0/9.9 ratio, with input images resized to 96 × 96 pixels, a resolution that matches the spatial granularity of DSSM cells and enables real-time perception within the computational budget typical of UAV-edge platforms.

Detector training and policy training are decoupled by design: the classifier is trained once on AIDERv2 and thereafter held frozen, and its measured error behaviour enters policy training through a calibrated stochastic error model instead of through per-step inference. This keeps the error process under experimental control and identical across configurations, seeds and ablations. The empirical confusion matrix obtained on the AIDERv2 test set instead serves as a stochastic noise model: the simulated detection output at cell c with ground-truth class $y^{*}\left(c\right)$ is drawn according to


$$\begin{array}{c} \hat{y}t\left(c\right)\text{ Categorical}\left({\hat{M}}_{y^{*}\left(c\right),:}\right) \end{array}$$ (1)

where $\hat{M}\in [0,1{]}^{K\times K}$ denotes the row-normalized empirical confusion matrix and ${\hat{M}}_{y^{*}\left(c\right),:}$ is the row corresponding to the true class. This offline characterization design constitutes a methodological choice that decouples detector training from policy training, enabling tractable closed-loop reinforcement learning at scale while preserving the empirical error structure of the trained detector.

The confusion matrix reproduces the marginal error rates of the trained detector but not the joint structure of its per-image confidence outputs, and it is therefore treated as a computational approximation rather than an unbiased substitute. The consequence of this approximation is examined empirically through replayed per-image predictions from the frozen detector and through controlled manipulation of the temporal persistence of detection errors, both reported among the detector-error analyses below.

### 2.4 The disaster situation state matrix

The DSSM aggregates per-observation detection outputs over time and supplies them to the scheduling policy as a fixed-size input. Evidence accumulates across repeated visits to the same cell, while exponential forgetting reflects the non-stationarity of the underlying disaster process. Compared with maintaining a full posterior over disaster states, this representation is structured, computationally lightweight, and of constant size regardless of how many cells have been observed. A structured and computationally lightweight alternative, the DSSM accumulates evidence across visits to the same cell while incorporating principled temporal forgetting to reflect non-stationarity of the underlying disaster process.

Formally, the DSSM at time t is a three-dimensional tensor $D_{t}\in [0,1{]}^{H\times W\times K}$, where H and W are spatial grid dimensions and K=4 is the number of disaster classes. The entry $D_{t}\left(c,k\right)$ encodes the current belief that cell c contains disaster class k, with the normalization constraint $\sum_{k}D_{t}\left(c,k\right)=1$ at every cell. Cells never observed hold the uniform prior, so that absence of evidence reaches the policy as uncertainty and not as a confident zero.

An update rule combines exponential temporal decay toward the uniform prior with assimilation of detection outputs at observed cells:


$$\begin{array}{c} D_{t}(c,k)=[\text{1}-\alpha (c)]\{\rho_{t}(c)D_{t-\text{1}}(c,k)+[\text{1}-\rho_{t}(c)]u_{\text{0}}(k\}+\alpha (c)\text{1}[\hat{y}(c)=k] \end{array}$$ (2)

Here $\alpha \left(c\right)=\alpha_{\text{0}}$ when cell c is observed at step t and α(c)=0 otherwise, $\alpha_{\text{0}}\in \left(0,1\right)$ being the assimilation weight applied to a fresh detection; $\rho_{t}\left(c\right)=e^{-\lambda \Delta t\left(c\right)}$ is the temporal persistence factor with decay rate λ=0.00505 per step and elapsed time Δt(c) since the previous update at that cell; $u_{\text{0}}\left(k\right)=1/K$ is the uniform prior; and 1[·] is the indicator function. For unobserved cells the rule reduces to $D_{t}(c,k)=\rho_{t}(c)D_{t-\text{1}}(c,k)+[\text{1}-\rho_{t}(c)]u_{\text{0}}(k)$, a passive relaxation toward the prior, and normalization preserves $\sum_{k}D_{t}\left(c,k\right)=1$ at every cell and step.

Fig 3 traces the update dynamics across consecutive steps, showing selective accumulation at observed cells alongside retention of the uniform prior at unobserved or stale cells.

**Fig 3:**
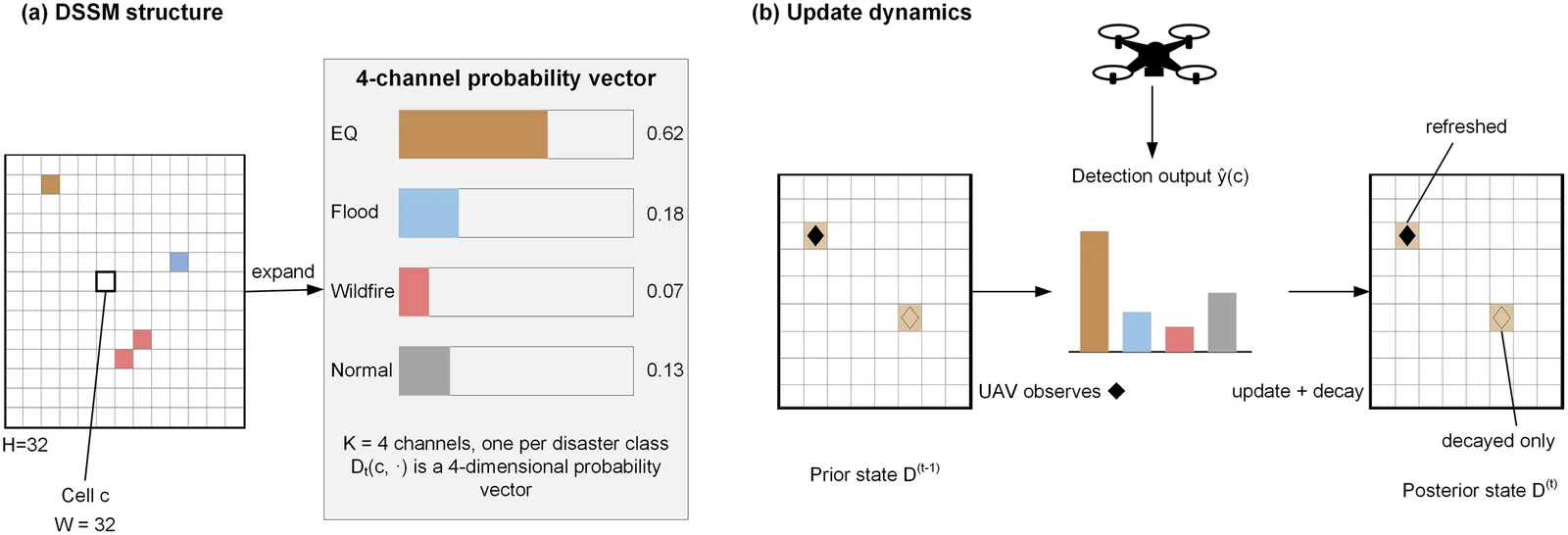
Structure and update mechanism of the Disaster Situation State Matrix.

### 2.5 PPO-based scheduling policy

Proximal Policy Optimization (PPO) trains the scheduling policy, with its clipped surrogate objective providing update stability in the high-variance setting of sequential dispatch [41]. PPO was selected over value-based alternatives such as DQN and over other actor-critic methods such as A2C because its on-policy updates track the DSSM state distribution as the closed loop shifts it, which off-policy methods cannot guarantee when the behaviour distribution drifts during training [42].

Observations at each step concatenate three components: the local DSSM view centered on the UAV’s current position with a 5 × 5 cell receptive field, a compact summary of the UAV’s own state comprising absolute coordinates and remaining battery, and a binary mask indicating which discovered demand points remain unserved. The composite observation combines local detail, on which immediate dispatch decisions depend, with global context, which informs planning across multiple demand points. The action space comprises the six discrete options (four cardinal directions, hover, and return-to-base), with the policy outputting a categorical distribution over these actions through a softmax head.

Configurations differ only in the composition of the first observation component. Where the state matrix is withheld from the policy, the local matrix view is removed while the auxiliary state vector and the mask of discovered but unserved demand points are retained, so that the withheld condition remains informed about which tasks exist and differs from the full condition only in its access to spatially resolved disaster belief, not in the availability of observations as such.

All four UAVs share a single policy network through parameter sharing and execute independently from their local observations, with implicit coordination emerging through the common DSSM that all UAVs read but only the observing UAV updates. A shared-trunk design underlies the network architecture. The DSSM convolutional branch comprises two 3 × 3 convolutional layers with 16 and 32 channels respectively, each followed by ReLU activation; the auxiliary-state branch comprises two fully-connected layers of 32 units each; the fused features pass through a shared MLP of two 64-unit hidden layers; and the actor and critic heads are single linear layers projecting to the action dimension (six) and a scalar value respectively. Fig 4 illustrates the architectural decomposition.

**Fig 4:**
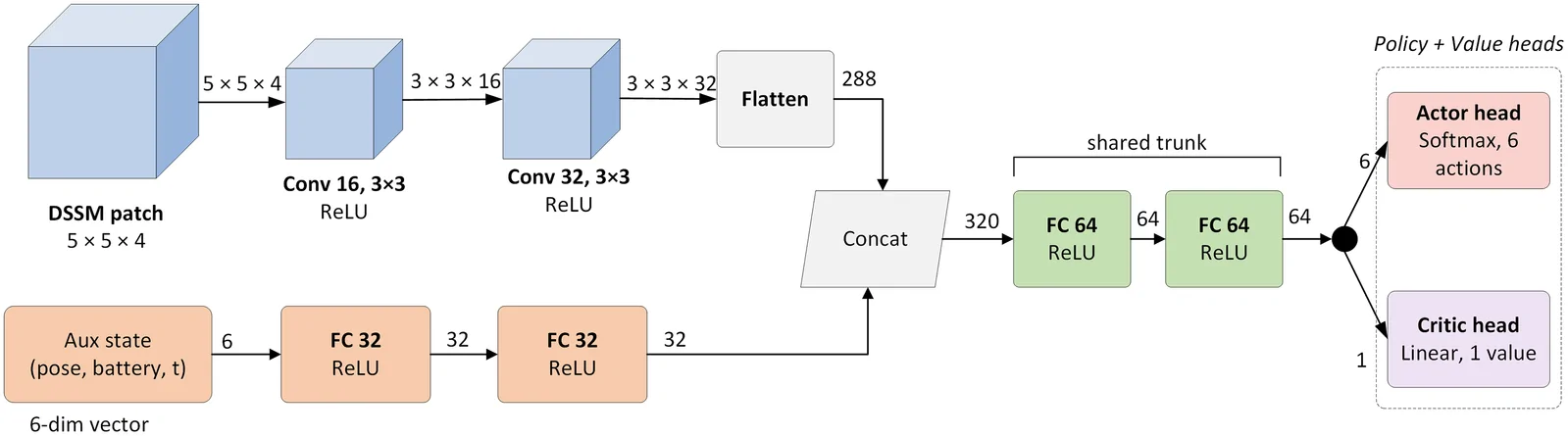
Network architecture of the DSSM-conditioned PPO policy, depicting the shared trunk and the parallel actor and critic heads.

### 2.6 Reward formulation and training procedure

The reward function comprises three additive terms corresponding to the principal operational objectives: timely service of affected demand points, low response latency, and economical use of flight resources. The instantaneous reward at step t is


$$\begin{array}{c} r_{t}=\alpha \cdot r_{t}^{\text{cov}}-\beta \cdot r_{t}^{\text{lat}}-\gamma \cdot r_{t}^{\text{eng}} \end{array}$$ (3)

where the coverage term $r_{t}^{\text{cov}}$ counts newly served demand points weighted by their disaster severity, the latency term $r_{t}^{\text{lat}}$ accumulates elapsed waiting time at all detected but unserved demand points, and the energy term $r_{t}^{\text{eng}}$ penalizes each unit of UAV motion. Default coefficients α=β=γ=1.0 are adopted for the principal experiments, with sensitivity examined through controlled ablations on reward weighting. Complete mathematical specification of each term is given in Table 2.

**Table 2:** Reward function decomposition with mathematical specification and operational rationale.

Term	Symbol	Mathematical form	Operational rationale
Coverage	$r_{t}^{\text{cov}}$	$\sum_{i\in {𝒮}_{t}^{\text{new}}}l_{i}$, where $l_{i}\in 1,2,3$ is severity of newly served demand point i	Encourages timely service of detected disaster zones, weighted by severity
Latency	$r_{t}^{\text{lat}}$	$\sum_{i\in 𝒰t}(t-\tau_{i}^{\text{detect}})/T_{max}$, where $\tau_{i}^{\text{detect}}$ is the detection time of unserved demand point i	Penalizes accumulated waiting time at detected demand points
Energy	$r_{t}^{\text{eng}}$	$\Delta d_{t}/d_{max}$, where $\Delta d_{t}$ is per-step cell displacement	Penalizes per-step UAV motion to encourage efficient routing

Table 2 documents the three reward components, their mathematical form, and the operational objective each addresses. This decomposition aligns with multi-objective reward designs adopted in recent disaster-response routing studies [43], with normalization choices ensuring each term contributes on a comparable numerical scale prior to weighting.

Training follows a multi-seed protocol. Each closed-loop configuration and learned algorithmic baseline is trained with ten independent random seeds (31–40), and every trained model is evaluated on one hundred frozen scenarios (2001–2100). Each training run executes 140,000 environment steps using four parallel environments. All runs use the same hyperparameters and save model-specific VecNormalize statistics, which are frozen during evaluation. The preceding description specifies hyperparameters and seed organization; the per-step interaction among detection, state aggregation, action selection and policy update is set out in Algorithm 1, where the four procedures correspond to the four stages of the loop and the configuration parameters isolate the two axes along which the ablations differ.

**Algorithm 1. Closed-loop training with perception-gated task discovery**

 **Require:** frozen detector $f_{\text{YOLO}}$ with empirical confusion matrix $\hat{M}\in [0,1{]}^{K\times K}$; assimilation weight $\alpha_{\text{0}}=0.7$; decay rate λ=0.00505; uniform prior $u_{\text{0}}=\frac{\text{1}}{K}1_{K}$; refresh interval κ∈{1,10,∞}; visibility flag ν∈{on,off}; PPO hyperparameters $\left(\varepsilon ,K_{\text{ep}},\gamma ,\zeta \right)$; episodes E; horizon T; vehicles N; grid H×W; classes K

 **Ensure:** trained scheduling policy $\pi_{\theta }$

1 initialize actor $\pi_{\theta }$, critic $V_{\phi }$; rollout buffer B←∅

2 for e=1,…,E do

3 reset environment; $D_{\text{0}}\leftarrow u_{\text{0}}⊗1^{H\times W}$; G←∅; G←∅ for all c

4 for t=0,…,T−1 do

5 $\langle {\hat{Y}}_{t},O_{t}\rangle \leftarrow Perceive\left(t\right)$

6 if t=0 or (κ≠∞ and tmodκ=0) then $D_{t}\leftarrow Update-DSSM\left(D_{t-1},{\hat{Y}}_{t},O_{t},t\right)$ else $D_{t}\leftarrow D_{t-1}$

7 for n=1,…,N do $Act\left(n,D_{t},G\right)$ end for

8 advance environment; update severities and release schedule

9 end for

10 PPO−Update

11 end for

12 return $\pi_{\theta }$

 **procedure** Perceive(t)

13 for n=1,…,N do $c_{n}\leftarrow$ position of vehicle n; draw $\hat{y}\left(c_{n}\right)\text{ Categorical}\left({\hat{M}}_{y\left(c_{n}\right),:}\right)$ end for

14 $O_{t}\leftarrow \{c_{n}{\}}_{n=1}^{N}$; ${\hat{Y}}_{t}\leftarrow \{\hat{y}(c):c\in O_{t}\}$

15 for $c\in O_{t}$ with $c\in O_{t}$ and c holding an unserved demand point do G←G∪{c} end for

16 return $\langle {\hat{Y}}_{t},O_{t}\rangle$

 **procedure** $Update-DSSM\left(D,\hat{Y},O_{t},t\right)$

17 for each cell c do

18  c; c; c

19 $D(c,k)\leftarrow [\text{1}-\alpha (c)]\{\rho_{t}(c)D(c,k)+[\text{1}-\rho_{t}(c)]u_{\text{0}}(k\}+\alpha (c)\text{1}[\hat{y}(c)=k]$ for k=1,…,K

20 $D(c,\cdot leftarrowD(c,\cdot )/\sum_{k}D\left(c,k\right)$; if $c\in O_{t}$ then
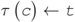


21 end for

22 return D

 **procedure** Act(n,D,G)

23 $x^{\left(n\right)}\leftarrow$ auxiliary state of vehicle n; $g^{\left(n\right)}\in \{0,1{\}}^{35}$ is the discovered-but-unserved demand mask

24 if ν=on then $P^{\left(n\right)}\in [0,1{]}^{5\times 5\times K}$ is the patch of D centred on $c_{n}$ and $s^{\left(n\right)}\leftarrow \langle P^{\left(n\right)},x^{\left(n\right)},g^{\left(n\right)}\rangle$ else $s^{\left(n\right)}\leftarrow \langle x^{\left(n\right)},g^{\left(n\right)}\rangle$

25 $a^{\left(n\right)}\pi_{\theta }(\cdot |s^{\left(n\right)})$ restricted to the feasible action mask; execute $a^{\left(n\right)}$; observe $r^{\left(n\right)}$

26 append $\left(s^{\left(n\right)},a^{\left(n\right)},r^{\left(n\right)}\right)$ to B

 **procedure** PPO−Update

27 compute generalized advantage estimates ${\hat{A}}^{\left(t\right)}$ from B with (γ,ζ)

28 update θ on $L^{\text{CLIP}}\left(\theta \right)$ and ϕ on $L^{V}\left(\phi \right)$ for $K_{\text{ep}}$ epochs; B←∅

Algorithm 1 separates the two axes varied in the ablations. The refresh interval κ governs how often line 6 admits new detection evidence, taking the value 1 for per-step refresh, 10 for the low-frequency condition, and∞for the initial-snapshot condition in which the matrix is formed once and thereafter held fixed. The visibility flagν governs the composition of the policy observation at line 24 and does not alter the maintenance of the matrix itself, so that the withheld conditions continue to update the representation while excluding it from the state on which actions are conditioned. Line 15 applies in every condition and to every baseline, making eligibility for service conditional on prior detection, not on ground-truth demand information. During evaluation the policy retains the sampling rule of line 25 with the random number state and the observation normalization statistics frozen, whereas the value-based baseline selects ${argmax}_{a}Q\left(s,a\right)$, and this distinction is preserved in all reported comparisons.

## 3 Results

### 3.1 Experimental protocol and evaluation metrics

Experimental evaluation comprises a complete two-by-two closed-loop ablation, matched algorithmic baselines, detector-error analyses, reward-function experiments and component-level timing. The primary outcomes are task completion rate, average response latency and cumulative flight distance, with false dispatches reported alongside the detector-error analyses. All compared conditions use the same frozen evaluation scenarios and identical hyperparameters, isolating the variable of interest in each comparison from confounded tuning effects.

Training seeds are the independent unit of inference. The one hundred frozen scenarios evaluated per model reduce the variance of each seed mean and are not treated as independent replicates. Paired differences are tested with the exact two-sided Wilcoxon signed-rank test, whose smallest attainable p-value at ten seeds is 0.001953, with paired rank-biserial correlation as the effect size, seed-level bootstrap confidence intervals, and Holm correction within each analysis family. Every reported result states the detection condition under which it was obtained, and results acquired under perfect detection and under the empirical detector are reported separately throughout.

### 3.2 Detection module baseline performance

The detector was trained on AIDERv2 and evaluated on the held-out test partition before integration into the closed loop, and the resulting per-class confusion pattern parameterizes the noise model used during policy training. The resulting empirical confusion matrix is reported in Fig 5.

**Fig 5:**
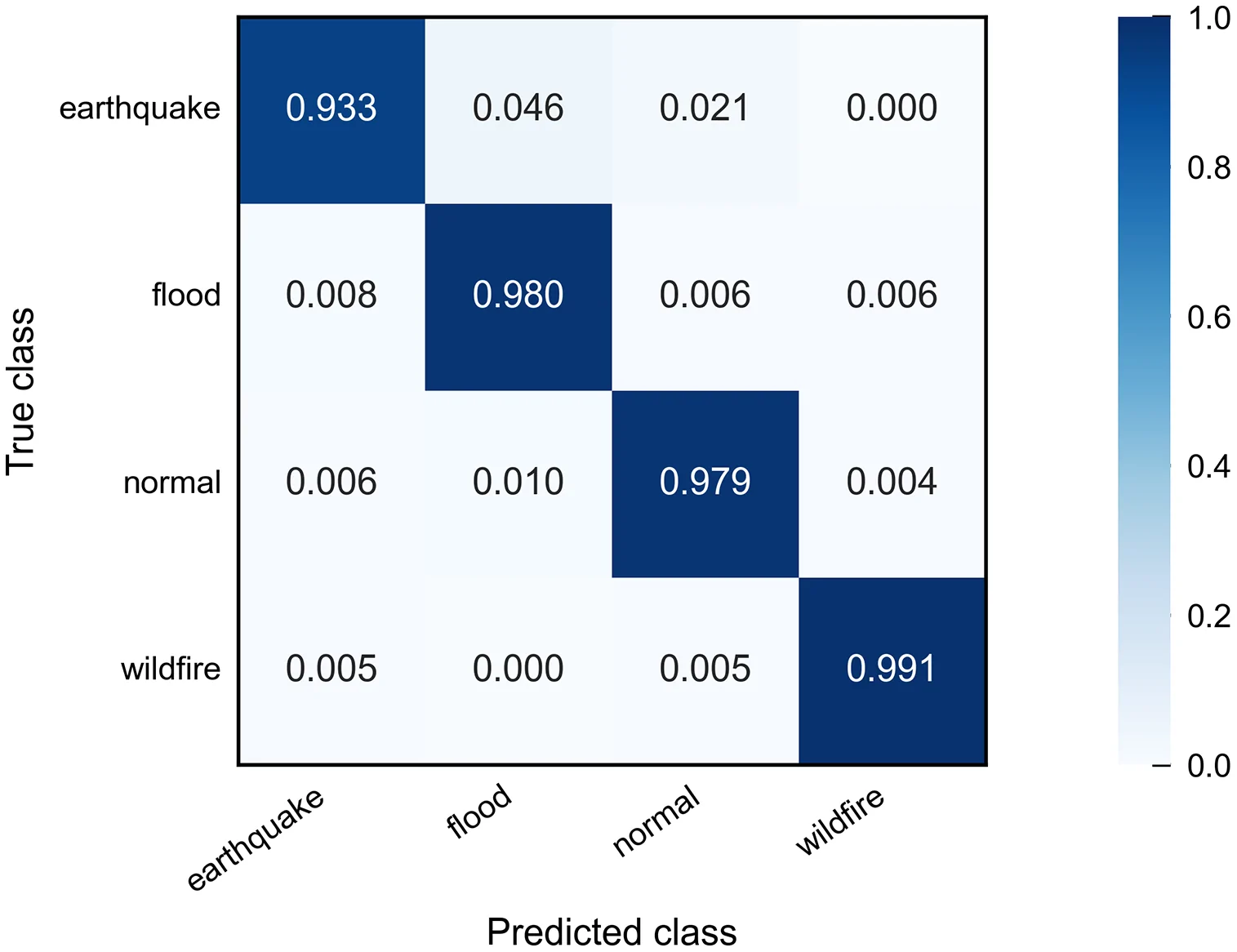
Disaster image recognition performance.

Fig 5 reports the frozen detector results on the held-out test partition. YOLOv8s attained 0.9758 test accuracy and 0.9725 macro-averaged $F_{\text{1}}$, with empirical false-negative and false-positive operating points of 0.010 and 0.021. The row-normalized confusion matrix provides the marginal error model used during policy training, while replayed per-image predictions are evaluated separately among the detector-error analyses.

The coupling gain is stable across detector capacity. Three detectors trained under an identical protocol reach test accuracies of 0.9680, 0.9758 and 0.9776 with macro-averaged $F_{\text{1}}$ of 0.9643, 0.9725 and 0.9750, and the completion advantage of the fully coupled configuration over the withheld configuration measures 35.85, 36.55 and 36.61 percentage points respectively, with pairwise differences whose intervals span zero. Across the accuracy range examined, the coupling effect does not depend on the capacity of the detector.

### 3.3 Closed-loop ablation analysis

The evaluated configuration set comprises C1, C2, C2b, C3 and C4. C1 uses only the initial DSSM snapshot. C2 updates the DSSM every step but withholds it from the policy, whereas C2b withholds the DSSM and refreshes it every ten steps. C3 exposes the DSSM to the policy with refresh every ten steps, and C4 exposes it with per-step refresh. All configurations retain the same perception gate, six-action interface, hyperparameters and seed organization.

Training curves under the empirical detector separate along the same axis as the evaluation outcomes (Fig 6). Configurations with policy access to the state matrix converge to a steady-state mean episodic reward of approximately 356 and 314, while the withheld configurations plateau near 58 and 53, and the initial-snapshot configuration settles near 290. The bulk of convergence completes within 50,000 environment steps in every learning configuration, and no configuration exhibits late-stage degradation over the training budget.

**Fig 6:**
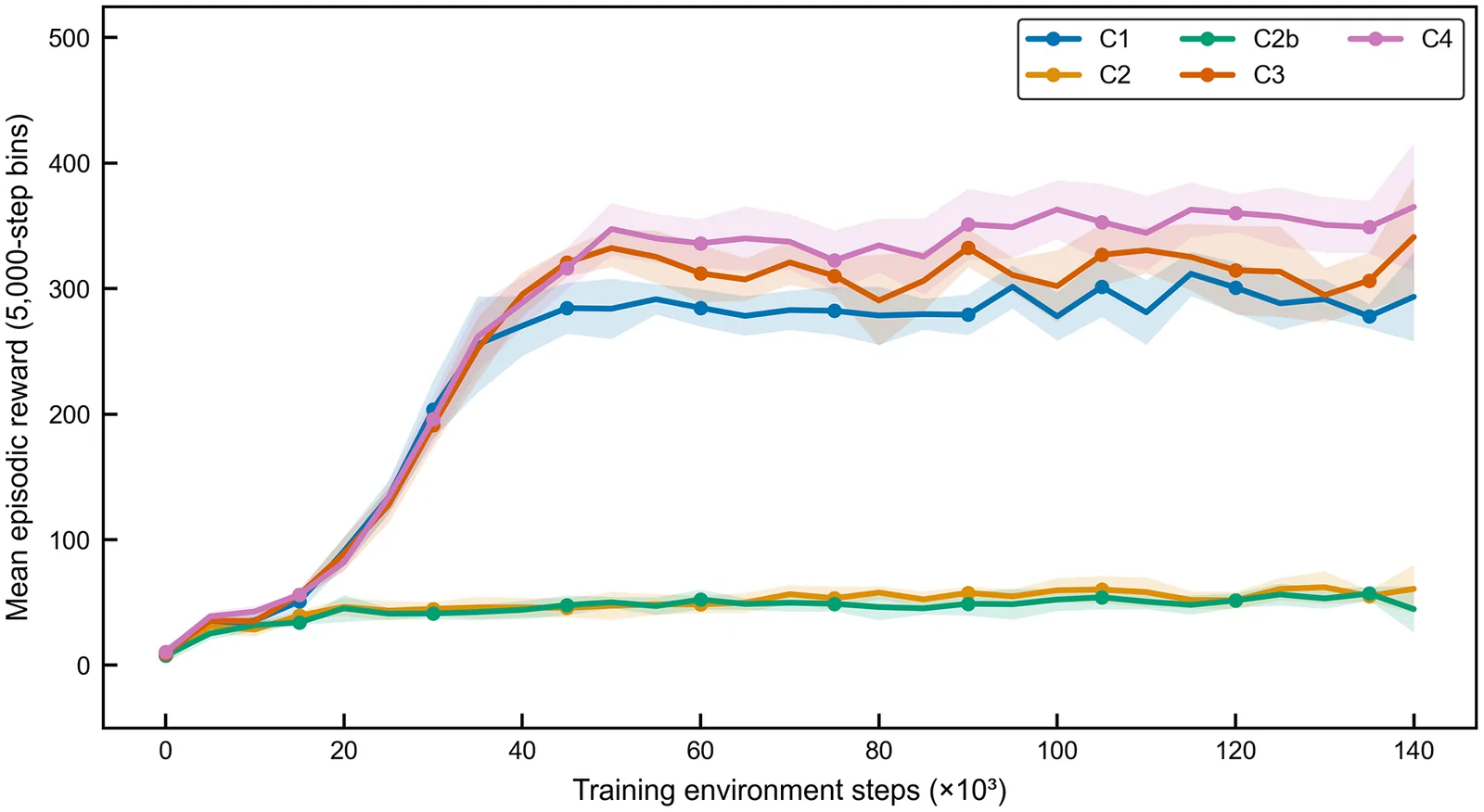
Training convergence across closed-loop configurations.

Trained policies are subsequently evaluated on one hundred held-out episodes per seed, with the resulting distributions of four primary metrics summarized in Fig 7.

**Fig 7:**
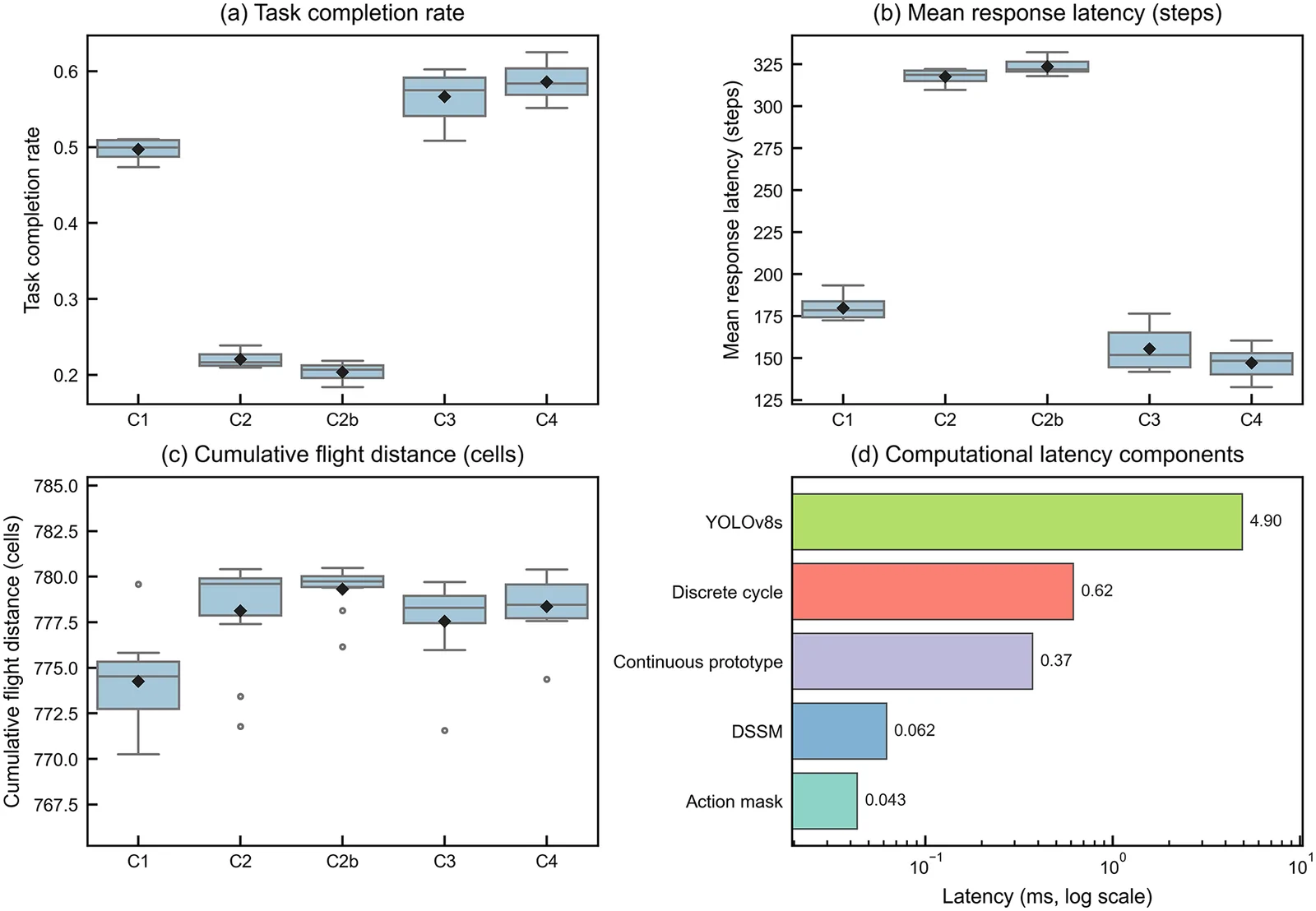
Evaluation metric distributions across closed-loop configurations, alongside the computational latency components of the proposed framework.

Task completion under the empirical detector separates the configurations into two regimes. Configurations in which the state matrix is withheld from the policy reach 0.2203 with per-step refresh and 0.2036 with refresh every ten steps, while configurations with policy access reach 0.5665 and 0.5859 respectively, and the initial-snapshot configuration reaches 0.4971. The factorial decomposition assigns a main effect of 36.42 percentage points to policy access (95% CI 34.83 to 37.79, Holm-adjusted p=0.0059) and 1.81 percentage points to refresh rate (95% CI 0.90 to 2.82, Holm-adjusted p=0.0195), with an interaction of 0.27 percentage points whose interval spans zero. Within the configurations that retain policy access, completion follows a monotone and diminishing progression across refresh regimes, rising by 6.94 percentage points from the initial snapshot to refresh every ten steps and by a further 1.94 percentage points at per-step refresh (Holm-adjusted p=0.084). The corresponding values under perfect detection are 0.5023, 0.2119, 0.2097, 0.5429 and 0.5811, and the difference between detection conditions at the fully coupled configuration is −0.48 percentage points with an interval spanning zero.

Statistical comparisons of each configuration against the fully closed-loop reference C4 are summarized in Table 3.

**Table 3:** Quantitative results and pairwise statistical comparisons for the five closed-loop configurations.

Configuration	Task completion (mean ± SD)	Seed-mean range	Response latency (mean ± SD)	Flight distance (mean ± SD)	Holm p vs C4	Rank-biserial
C1	0.497 ± 0.013	0.473–0.510	179.8 ± 6.7	774.3 ± 2.6	0.0078	−1.000
C2	0.220 ± 0.011	0.209–0.239	317.5 ± 4.5	778.1 ± 3.0	0.0078	−1.000
C2b	0.204 ± 0.012	0.184–0.219	323.6 ± 4.6	779.3 ± 1.3	0.0078	−1.000
C3	0.566 ± 0.032	0.508–0.602	155.5 ± 12.4	777.5 ± 2.4	0.0840	−0.636
C4	0.586 ± 0.024	0.551–0.625	147.0 ± 8.7	778.4 ± 1.7	—	—

C4 exceeds C1 by 8.87 percentage points (95% CI 7.26 to 10.32, Holm-adjusted p=0.0078).

### 3.4 Comparison against algorithmic baselines

Four baselines and the proposed scheduler hold the coupling fixed at the C4 configuration and vary only the scheduling policy, separating the contribution of the learning algorithm from that of the closed-loop architecture. B1 is a uniformly random policy; B2 is a greedy heuristic dispatching the nearest detected demand point; B3 substitutes DQN for the PPO policy network; B4 substitutes A2C; and B5 is the proposed PPO scheduler. Performance distributions, statistical comparisons against B5, and the corresponding effect sizes are summarized in Table 4.

**Table 4:** Performance and statistical comparison of the four algorithmic baselines and the proposed PPO scheduler.

Baseline	Task completion (mean ± SD)	Seed-mean range	Response latency (mean ± SD)	Flight distance (mean ± SD)	Holm-adjusted p vs PPO	Rank-biserial
B1 Random	0.095 ± 0.045	0.000–0.229	374.3 ± 17.8	770.1 ± 1.3	0.0078	−1.000
B2 Greedy	0.551 ± 0.035	0.457–0.657	155.6 ± 13.6	272.2 ± 63.4	0.0078	−1.000
B3 DQN	0.408 ± 0.120	0.180–0.543	230.5 ± 54.8	468.8 ± 230.4	0.0078	−1.000
B4 A2C	0.255 ± 0.009	0.242–0.271	308.8 ± 3.8	770.0 ± 2.2	0.0078	−1.000
B5 PPO (Ours)	0.586 ± 0.024	0.551–0.625	147.0 ± 8.7	778.4 ± 1.7	—	—

Holding the perception constraint, action interface and evaluation scenarios fixed, the proposed scheduler attains 0.5859 against 0.5514 for the greedy heuristic, an advantage of 3.44 percentage points (95% CI 2.03 to 4.91, Holm-adjusted p = 0.0078). The margin over the value-based and synchronous actor-critic alternatives measures 17.75 and 33.09 percentage points respectively (Holm-adjusted p = 0.0078 in both comparisons), placing those baselines at 0.4084 and 0.2550. Every policy in this comparison operates under the same detection gate, so that differences reflect the scheduling rule alone. The greedy heuristic reaches its completion level at a markedly lower cumulative flight distance, 272.2 cells against 778.4, because it halts once locally reachable demand points are exhausted, whereas the learned scheduler continues to expend the remaining battery budget on coverage. The two policies therefore differ in how a fixed energy budget is allocated as well as in completion, and the energy weight γ is the parameter that controls this allocation.

Quantitative differences between policies are mirrored qualitatively in the spatial behavior of the resulting dispatch trajectories, which are visualized for three representative configurations spanning the heuristic-to-closed-loop range in Fig 8.

**Fig 8:**
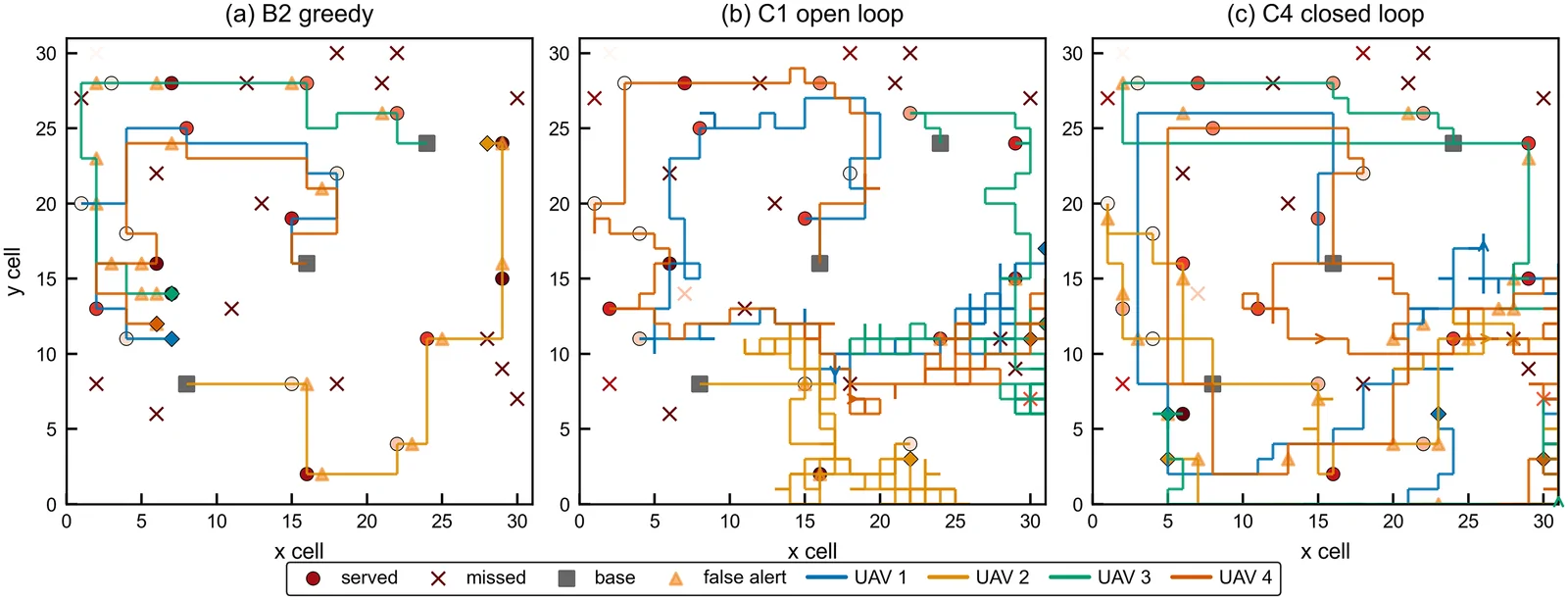
Representative UAV dispatch trajectories with disaster severity background and per-cell outcome markers.

Fig 8 overlays representative trajectories of the four vehicles onto the disaster severity field and marks per-cell outcomes as served, missed, or false alarm. The greedy trajectory concentrates near the region of initial deployment, the initial-snapshot trajectory disperses more widely while leaving cells in the lower-left and upper-right quadrants unserved, and the fully coupled trajectory yields the highest density of served cells at a comparable cumulative flight distance.

### 3.5 Robustness to detection noise

Robustness to detection noise is examined through two complementary analyses: a synthetic noise injection procedure that sweeps three categories of error at controlled levels, and a real-noise calibration procedure that maps the empirical YOLOv8 confusion matrix onto the synthetic noise coordinate system. The synthetic sweep proceeds by perturbing the row distributions of $\hat{M}$ in three structurally distinct ways: false negative injection that converts disaster cells to normal classifications with probability p, false positive injection that converts normal cells to randomly sampled disaster classifications with probability p, and localization error that displaces the position of disaster cells by Gaussian-distributed offsets with standard deviation σ. Resulting performance trajectories across noise categories and levels are reported in Fig 9.

**Fig 9:**
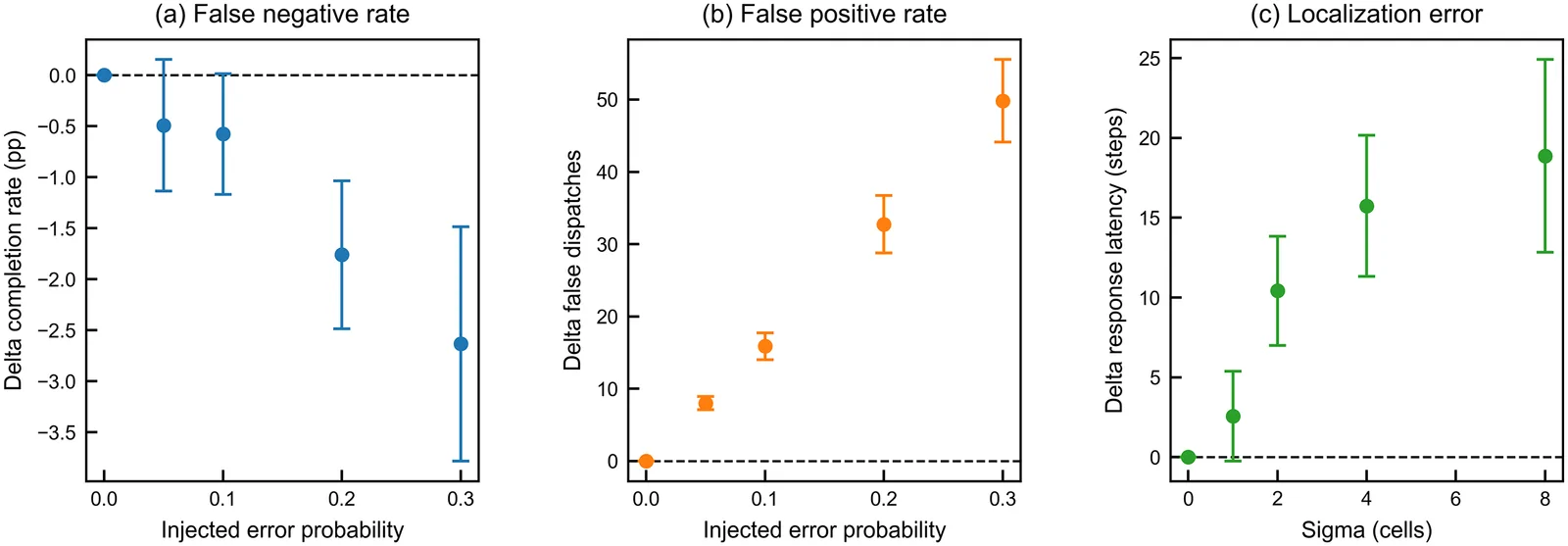
Seed-paired sensitivity of completion rate, false dispatches and response latency to three categories of synthetic detection error.

Fig 9 shows seed-paired means and 95% confidence intervals for the three synthetic-error families. False-negative rates of 0.20 and 0.30 reduce completion by 1.76 and 2.63 percentage points respectively (Holm-adjusted p=0.0234). At a false-positive rate of 0.30, false dispatches increase by 49.83, while localization errors with σ=2, 4 and 8 increase response latency by 10.42, 15.75 and 18.88 steps. The three error families therefore surface in distinct operational outcomes and do not collapse onto one common degradation curve.

Mapping of real YOLOv8 errors onto these synthetic noise curves provides the central calibration linking detector performance to system-level outcomes, with results reported in Fig 10.

**Fig 10:**
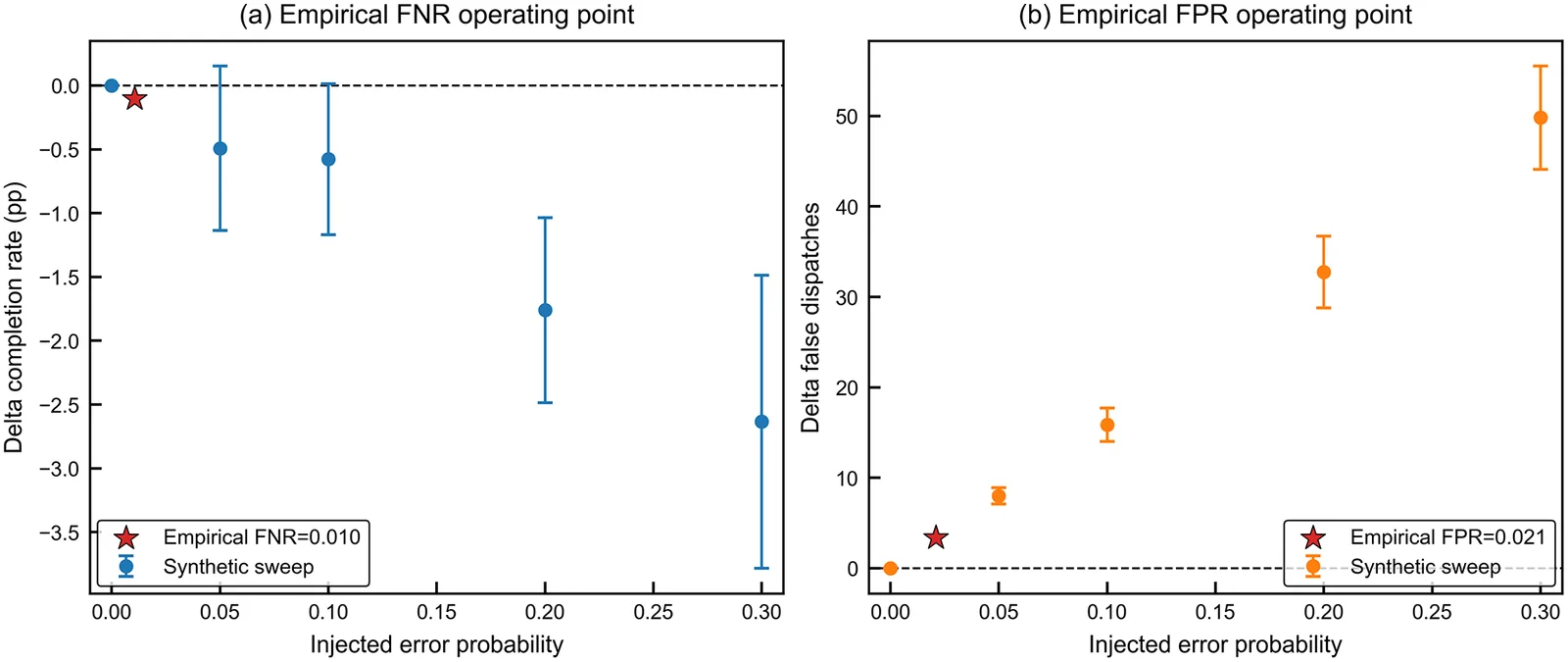
Calibration of real detection error against synthetic noise curves.

Fig 10 locates the empirical YOLOv8s false-negative rate of 0.010 and false-positive rate of 0.021 on the corresponding synthetic-error axes. These operating points serve as calibration references only; they are not evidence that confusion-matrix sampling reproduces the joint or temporal structure of per-image prediction errors.

Two manipulations examine whether the offline error model preserves the operational consequences of detection error. Replaying stored per-image predictions from the frozen detector in place of confusion-matrix sampling lowers completion from 0.5859 to 0.5731, a small but statistically resolved reduction of 1.28 percentage points (95% CI −2.26 to −0.35), indicating that independent confusion-matrix sampling is mildly optimistic relative to replayed predictions. Introducing temporal persistence into detection errors widens the separation: at a retention probability of 0.9 completion falls to 0.5581, a reduction of 2.77 percentage points (Holm-adjusted p=0.0059), while the longest uninterrupted run of retained errors extends from a single step to seventy-one steps and the observed miss rate rises from 0.93 to 6.59 percent.

### 3.6 Reward function ablation, weight sensitivity, and computational cost

Two questions concern the reward function: whether each term contributes a non-redundant learning signal, and whether performance is robust to perturbation of the weights around their defaults. Reward term ablation removes each component individually by setting its weight to zero and retraining from scratch under identical conditions, while weight sensitivity perturbs each weight independently to a higher or lower value with all three terms retained. Both analyses share the protocol of ten independent training seeds and one hundred evaluation episodes, with results summarized in Table 5.

**Table 5:** Reward function ablation and weight sensitivity.

Setting	Task completion (mean ± SD)	Response latency	Flight distance
Full	0.586 ± 0.024	147.0 ± 8.7	778.4 ± 1.7
w/o coverage	0.000 ± 0.000	410.1 ± 0.0	1.0 ± 2.7
w/o latency	0.597 ± 0.019	143.1 ± 6.7	776.9 ± 3.3
w/o energy	0.583 ± 0.026	148.6 ± 11.0	779.7 ± 0.8
high coverage	0.588 ± 0.037	146.3 ± 14.8	778.4 ± 2.7
high latency	0.584 ± 0.031	147.9 ± 12.9	777.8 ± 3.6
high energy	0.600 ± 0.049	142.7 ± 17.8	776.2 ± 3.3
low all	0.606 ± 0.015	140.2 ± 6.9	776.0 ± 5.2

Removing the coverage term collapses completion by 58.58 percentage points (Holm-adjusted p=0.0137), identifying it as the dominant learning signal. Removing the energy or the latency term changes completion by −0.32 and +1.15 percentage points respectively, with intervals spanning zero, and the same pattern holds under a reduced battery budget and under doubled severity growth, where removal of the corresponding term shifts completion by −0.60 and −0.71 percentage points with intervals spanning zero. The two auxiliary terms therefore operate as weak shaping signals over the range of conditions examined, and dispatch performance is insensitive to their weighting. Augmenting the state matrix with a normalized-entropy channel raises completion from 0.5859 to 0.6080, a difference of 2.21 percentage points whose interval spans zero (95% CI −0.65 to 4.79), establishing the direction of the effect without resolving its magnitude.

Computational efficiency of the closed-loop framework, displayed in the right-most panel of Fig 7, is quantified in Table 6. Measurements use an Intel i5-12400F, an NVIDIA GTX 1050 Ti, PyTorch 2.5.1, CUDA 12.1, FP32 and batch size one; every component is evaluated after 100 warm-up calls over 1,000 synchronized timed calls.

**Table 6:** Computational latency of the closed-loop framework on the reference hardware.

Component	Latency (ms)	Note
DSSM update	0.062	Single-cell probability update
Six-action feasibility mask	0.043	Physical action validity
Discrete PPO policy decision	0.434	Mean of 1000 policy calls under the evaluation sampling rule
Discrete scheduling cycle	0.615	DSSM update + mask + policy
Continuous scheduling/control prototype	0.373	Action head + simple kinematics + obstacle projection
YOLOv8s classification inference	4.905	1000 predictions, imgsz = 96, batch = 1, FP32

Component-level timing on the reference desktop hardware places the state-matrix update at 0.062 ms, the action-feasibility mask at 0.043 ms, the discrete policy forward pass at 0.434 ms and the complete discrete scheduling cycle at 0.615 ms, against 4.905 ms for single-image detection, giving a component-level total near 5.5 ms per vehicle. A continuous action head with simple kinematic integration and vectorized dynamic-obstacle projection completes in 0.373 ms, indicating that continuous action computation is not the limiting term. These measurements characterize the scheduling and perception code paths in isolation and exclude aerodynamic modelling, communication, sensor input and output, trajectory optimization and certified flight control, and they are not extended here to a complete airborne system.

## 4 Discussion

The separation between configurations that expose the state matrix to the policy and those that do not accounts for the largest share of the observed variation, while the rate at which that matrix is refreshed contributes a smaller and monotone increment that diminishes as refresh becomes more frequent. Structural coupling rather than information currency is therefore the operative mechanism, and the ordering of configurations tracks the conditional pattern of access, not the volume of information generated. This reading is consistent with formulations in which the value of a learned policy derives from what the state representation makes available to the decision and not from the fidelity of the underlying estimate, as reported for maintenance policies learned over partially observable states that resist direct measurement [30]. It also qualifies a common premise of scheduling studies that supply the environment state directly to the policy [22,27]: where perception governs which tasks become actionable, the representation that mediates between sensing and decision is not an implementation detail but a determinant of attainable performance. The magnitude of the refresh contribution further indicates that a system already exposing a decayed spatial belief to its scheduler gains comparatively little from additional update rate, which bears on edge deployments where update frequency competes with other computational demands [9,10]. The result also places dispatch scheduling within the scope of active perception, where the informational consequence of an action is treated as part of its value [34]. The two-by-two decomposition indicates which half of that proposition carries the effect in this setting: exposing the aggregated perceptual state to the policy accounts for the bulk of the observed variation, whereas increasing the rate at which that state is refreshed contributes comparatively little once exposure is in place.

Detection errors have measurable but outcome-specific downstream costs. False negatives reduce completion, false positives increase unnecessary dispatches, and localization error increases response time. Replayed per-image predictions yield lower completion than independent confusion-matrix sampling, while strongly persistent errors produce the largest additional decrease, and the coupling gain remains stable across detector capacity. The three detectors examined span a narrow accuracy range of 0.968 to 0.978, so this stability characterizes behaviour within that range and does not extend to detectors at substantially lower accuracy, where the dependence of the coupling gain on detector quality lies outside the scope of the measurements reported here. Aggregate detector accuracy alone therefore does not characterize the operational effect of an error process; its type and temporal duration must also be considered. Detection research has pursued accuracy under adverse viewing conditions through architectural refinement and complementary sensing [17,20,21], and reviews of aerial recognition identify class imbalance and domain shift as persistent constraints on that trajectory [19,44]. The present results locate a further dimension: two detectors matched on aggregate accuracy need not be matched on the duration of the error episodes they produce, and it is that duration to which the scheduling outcome responds. Evaluations of perception intended for closed-loop use accordingly benefit from reporting the temporal structure of errors alongside conventional accuracy measures, a requirement that systems-level assessments of humanitarian robotics have raised in more general terms [45].

The error model applied during policy training samples independently across observations, and the comparison against replayed per-image predictions and against varying temporal persistence quantifies the consequence of that simplification. The remaining simplifications concern the environment, not the error model. Motion is restricted to four cardinal directions on a two-dimensional lattice with a static road graph and one disaster class per cell, an abstraction that sets aside the aerodynamic interaction and continuous trajectory control central to flight in unconstrained environments [46], and one that omits the multi-modal communication constraints arising in cooperative search and rescue [47]. The results reported here are correspondingly not extended to continuous control, aerodynamic interaction, or spatially overlapping disaster types.

Follow-up research will progressively relax these simplifications. Reinforcement-learning policies attaining competitive performance against expert human operators in physical flight [48] provide existence proof that simulation-trained policies can migrate to deployment once the action interface is bridged to continuous control, while goal-conditioned decomposition of long-range planning from low-level control offers one route by which a discrete dispatch policy might retain its structure under such a bridge [12]. Extension of the environment to three-dimensional disaster diffusion with multi-class spatial overlap, and of the perception module to detectors characterized by error duration as well as aggregate accuracy, would together establish whether the coupling effect measured here persists under conditions closer to field operation.

## 5 Conclusion

This study measured the contribution of bidirectional perception-action coupling in unmanned aerial vehicle emergency response scheduling under a shared action interface and perception-gated task discovery. Runtime perception updating raised task completion by 8.87 percentage points over an initial-snapshot policy, and a complete factorial ablation attributed 36.42 percentage points to policy access to the disaster state matrix against 1.81 percentage points to refresh rate, with no detectable interaction between the two. Under identical constraints the learned scheduler exceeded a greedy heuristic by 3.44 percentage points. Detection error acted on performance principally through the duration of error episodes and only secondarily through marginal error rate, with replayed per-image predictions and temporally persistent errors each reducing completion relative to independent confusion-matrix sampling.
